# Supplementary material for: The effects of genital myiasis on the diversity of the vaginal microbiota in female Bactrian camels
Source: BMC Vet Res. 2022 Mar 5;18:87. doi: 10.1186/s12917-022-03189-5 (PMC8897907; doi:10.1186/s12917-022-03189-5)

pdfmake (since it's based on pdfkit) supports JPEG and PNG format
If no width/height/fit is provided, image original size will be used

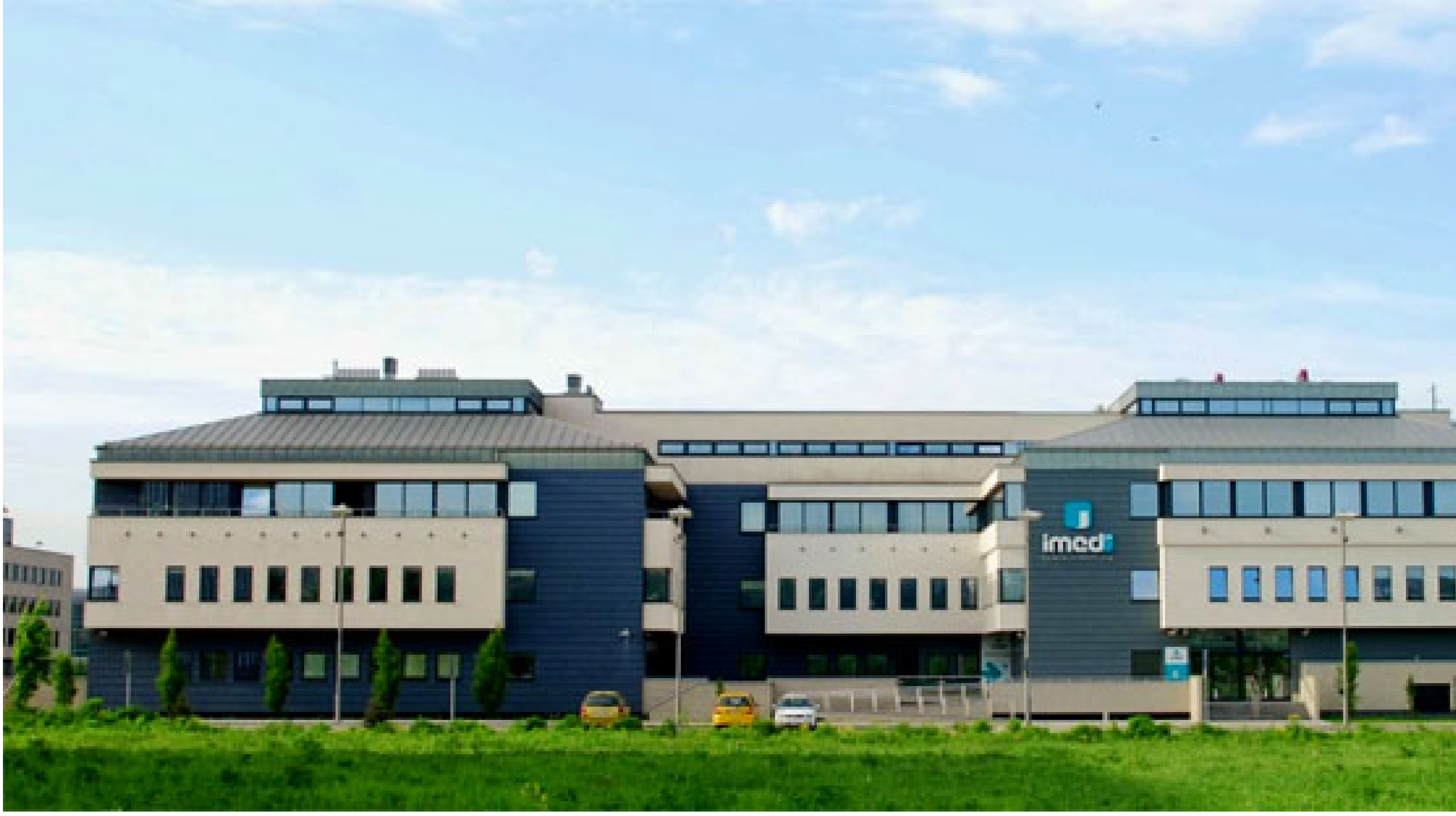

If you specify width, image will scale proportionally

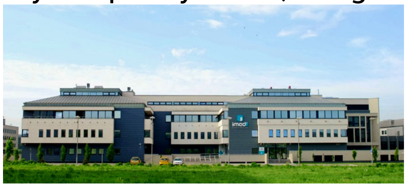

If you specify both width and height - image will be stretched

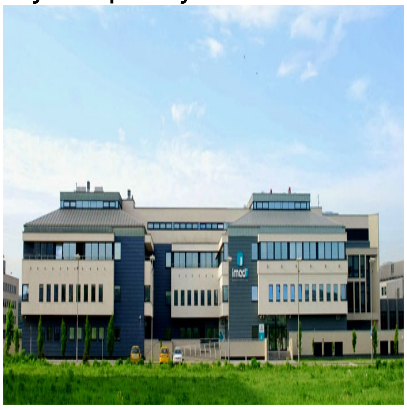

You can also fit the image inside a rectangle

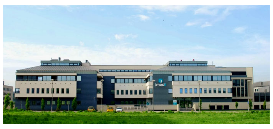

Images can be also provided in dataURL format
(the one below was taken from http://www.clipartbest.com/clipart-dT7zx5rT9)

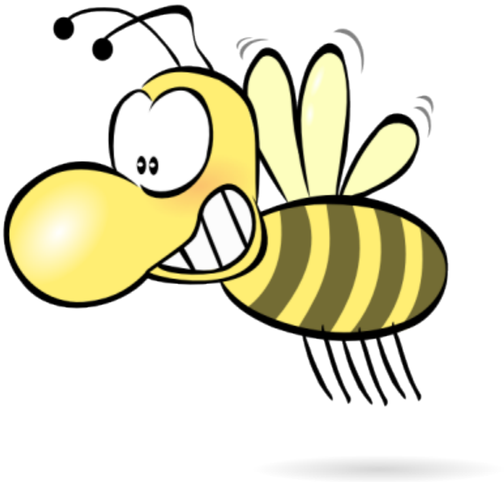

or be defined in the "images" dictionary, which can be referenced by name:

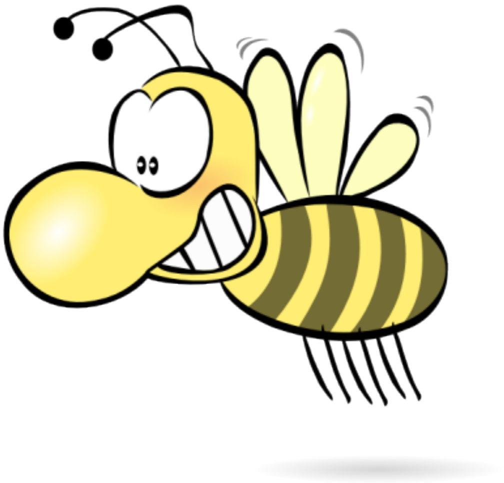

Supplement: Supplementary file 5 — Additional file 5. [file 12917_2022_3189_MOESM5_ESM.zip › MPL201709200_16s_yy/Treat1/css/js_css/pdfmake-master/examples/pdfs/images.pdf]
